# Periprosthetic breast capsules and immunophenotypes of inflammatory cells

**DOI:** 10.1007/s00238-012-0728-9

**Published:** 2012-05-17

**Authors:** Maria Elsa Meza Britez, Carmelo Caballero LLano, Alcides Chaux

**Affiliations:** 1 Department of Surgery, Clinical Hospital, Facultad de Ciencias Medicas, Universidad Nacional de Asuncion, Asuncion, Paraguay; 2 Department of Pathology, Instituto Nacional del Cancer, Aregua, Paraguay; 3 Department of Laboratory Research, Universidad del Norte, School of Medicine, Research Laboratory, Asuncion, Paraguay

**Keywords:** Periprosthetic breast capsule, Capsular contracture, Inflammatory cells, Immunophenotype, Antibodies, Breast implant

## Abstract

**Background:**

Silicone gel-containing breast implants have been widely used for aesthetic and reconstructive mammoplasty. The development of a periprosthetic capsule is considered a local reparative process against the breast implant in which a variety of inflammatory cells may appear. Nevertheless, only few reports have evaluated the immunophenotypes of those inflammatory cells. Herein, we aim to provide more information in this regard evaluating 40 patients with breast implants.

**Methods:**

We studied the immunophenotype of the inflammatory cells of capsular implants using antibodies against lymphocytes (CD3, CD4, CD8, CD20, CD45, and CD30) and histiocytes (CD68). Percentages of CD3 and CD20 positive cells were compared using the unpaired Student's *t* test. Fisher's test was also used to compare Baker grades by implant type, implant profile, and location and the presence of inflammatory cells by implant type.

**Results:**

The associations between Baker grades and implant type and location were statistically nonsignificant (*p* = 0.42 in both cases). However, the use of low profile implants was significantly associated (*p* = 0.002) with a higher proportion of Baker grades 3 and 4. We found evidence of inflammation in 92.5 % of all implant capsules, with a statistically significant (*p* = 0.036) higher proportion in textured breast implants. T cells predominated over B cells. Textured implants elicited a more marked response to T cells than smooth implants, with a similar proportion of helper and cytotoxic T cells. Textured implants showed statistically significant higher percentages of CD3 positive cells than smooth implants. Percentages of CD20 positive cells were similar in textured and smooth implants.

**Conclusions:**

These results suggest that textured breast implants might induce a stronger local T cell immune response. Our findings could shed some light to understand the association of silicone breast implants and some cases of anaplastic large cell lymphomas.

Level of Evidence: Level III, prognostic study.

## Introduction

Silicone gel-containing breast implants have been widely used for aesthetic and reconstructive mammoplasty. The development of a periprosthetic capsule is considered a local reparative process against the breast implant in which a variety of inflammatory cells may appear. Nevertheless, only few reports have evaluated the immunophenotypes of those inflammatory cells. Herein, we aim to provide more information in this regard evaluating 40 patients with breast implants [1–3]. Our study focused on capsules with and without capsular contracture, including replacement of round textured and smooth breast implants in aesthetic surgery [4]. Characteristics of inflammatory cells are reported in the present study, along with the association between lymphocytic and histiocytic immunophenotypes and clinicopathologic features.

## Materials and methods

This is a prospective study of periprosthetic breast capsules from 40 consecutive patients (or 80 capsules) with replacement of gel-containing breast implants for aesthetic augmentation between March 2008 and December 2010. Capsular contracture was graded following the Baker scale method, ranging from 1 to 4. The new implant type was chosen by the patient and the plastic surgeon based on preoperative breast size and chest dimensions. Gel-containing breast implants were replaced regardless of type (textured or smooth) or profile (high or low). In each case, the anesthesia was either general or thoracic epidural [5, 6]. A periareolar approach was carried out to remove the previous implant. In all cases, the new implant was smooth, round, and of high profile, placed in a submuscular location. A liquid sample of the virtual capsular space was taken for microbiologic culture. The entire capsular implant or a representative fraction of it was fixed in 10 % buffered formalin and sent for pathologic analysis. A negative vacuum drain left during the surgery was removed at 4th or 5th postoperative day depending on the quantity and quality of the liquid drained. Antibiotics were used in each patient as follows: 1 g of cefazolin before surgery, 1 g of cefazolin before introducing the implants, and a final dose of cefaloxim 1 g 8 h after surgery [7, 8]. Also, mild thoracic compression with bandage was used to cover the surgical area.

Hematoxylin and eosin-stained slides were prepared from routinely processed capsular biopsies or excision specimens. For immunohistochemical stains, sections were cut at 3 μm and mounted on silane-coated slides. Sections were deparaffinized and rehydrated. Endogenous peroxidase activity was blocked by 3 % hydrogen peroxide for 10 min. Antigen retrieval was performed in 10 mM sodium citrate buffer (ph 6) for 15 min. Tissue sections were incubated with IgG monoclonal antibodies directed against CD3, CD4, CD8, CD20, CD45, CD30, and CD68 (1 h at room temperature). After incubation, specimens were washed with phosphate buffered saline (PBS)-tween buffer. The secondary biotinylated antibody was applied, followed by the streptavidin–biotin–peroxidase complex. Samples were then washed with PBS-tween buffer and incubated with freshly prepared diaminobenzydine (DAB) + substrate–chromogen buffer at room temperature. After gently rinsing with distilled H_2_O, slides were counterstained with hematoxylin and mounted with permanent media. Both positive and negative controls were included for each immunohistochemical batch.

### Statistical analysis

Percentages of CD3 and CD20 positive cells were compared using the unpaired Student's *t* test. Baker grades were compared by implant type, implant profile, and location using the Fisher's exact test. Fisher's test was also used to compare presence of inflammatory cells by implant type. A two tailed *p* < 0.05 was required for statistical significance. Data were analyzed using STATA release 11 (StataCorp Inc., College Station, TX, USA).

## Results

**Fig. 1:**
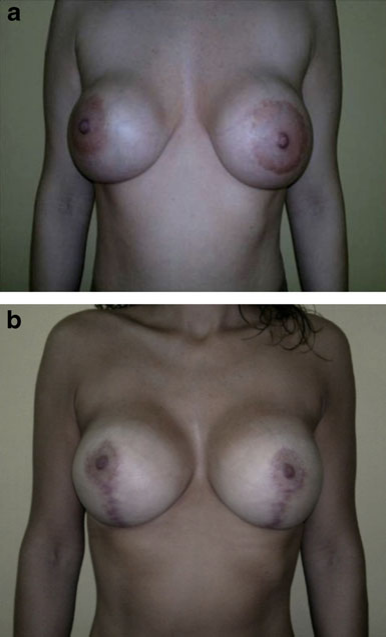
**a** Female, 35 year-old, preoperative view, with capsular contracture, Baker IV. **b** postoperative view, 7 months after breast surgery

**Fig. 2:**
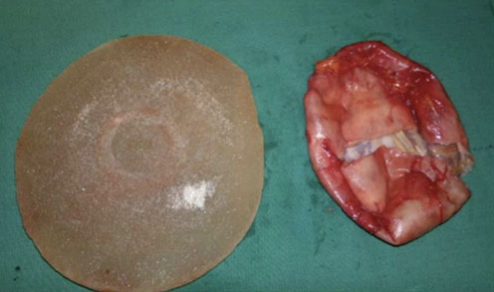
Breast textured implant with periprosthetic capsule

All patients were females with an age range of 22 to 45 years (median 36.5 years). All patients were healthy without aggravating factors except one patient who had a mycobacterium infection following her first breast implant. In 25 cases, the presence of contracture was the cause of the replacement (Fig. 1). In the remaining 15 cases, the patient chose a larger size for the breast implant. The implants were textured in 35 cases and smooth in the remaining five cases (Fig. 2). All textured implants were subglandular while the smooth implants were submuscular. Baker grades 1 and 4 were the most frequent, with 15 and 16 cases, respectively. Baker grade 3 was documented in two cases and Baker 3 grade in the remaining seven cases. The associations between Baker grades and implant type and location were statistically nonsignificant (*p* = 0.42 in both cases). However, the use of low profile implants was significantly associated (*p* = 0.002) with a higher proportion of Baker grades 3 and 4 (Table 1). No bacterial growth was observed in the cultures, even in the case with a previous mycobacterium infection.

**Table 1:** Association of capsular profile (High vs. Low) and capsular contracture by Baker grades

Baker contracture	High proflie (%)	Low proflie (%)	Total
Grade 1	15 (100)	0 (0)	15
Grade 2	2 (100)	0 (0)	2
Grade 3	3 (42.9)	4 (57.1)	7
Grade 4	8 (50)	8 (50)	17

*P* (Fisher’s exact) = 0.002

**Fig. 3:**
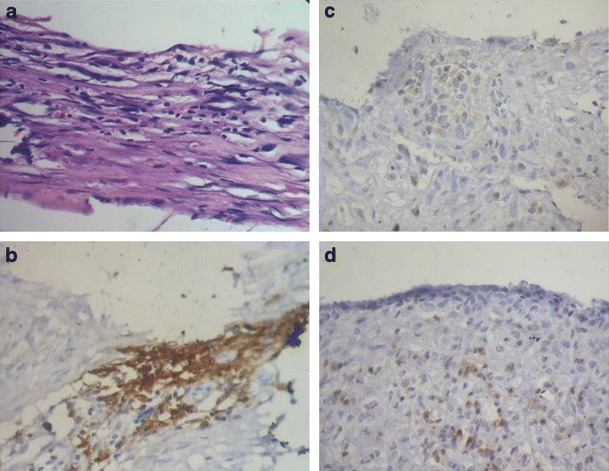
**a** Hematoxylin and eosin (H & E) section showing capsular inflammatory infiltrate (original magnification × 200); b with predominant cd3 + T cell; c and a similar proportion of cd4 + T-helper; d and cd8 T-cytotoxic cells (*brown stain* in **b**, **c**, and **d**)

**Fig. 4:**
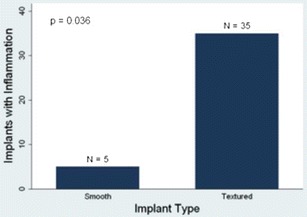
Presence of inflammation and type of breast implant. An inflammatory response was more frequent in textured implants

In 37 cases, the pathology study showed inflammatory infiltrate in the implant capsule (Fig. 3). Among the cases with inflammation, three of five (60 %) of the implants were smooth, and 34 of 35 (97 %) were textured. The association of inflammatory cells with implant type was statistically significant (*p* = 0.036, Fig. 4). Moreover, in 16 cases, we observed focal presence of granulomas (siliconomas); all of which but one occurred in textured implants. Lymphocytes predominated, with a minor histiocytic component.

**Fig. 5:**
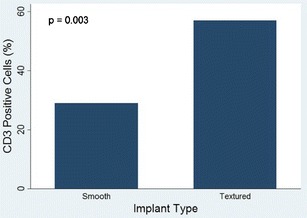
CD3 positive cells and type of breast implant. CD3-positive cells were more prevalent in textured implants

**Fig. 6:**
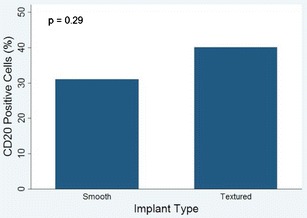
CD20-positive cells and type of breast implant. Proportions of CD20-positive cells were similar in smooth and textured implant

The immunohistochemistry showed a predominance of T cells over B cells (58 vs. 42 %). Textured implants elicited a more marked response to T cells than smooth implants, with a similar mean proportion of CD4 and CD8 positive cells (48 and 52 %, respectively). Textured implants showed statistically significant higher mean percentages of CD3 positive cells than smooth implants (57 vs. 29 %, *p* = 0.003, Fig. 5). The mean percentages of CD20 positive cells were similar in textured and in smooth implants, and the difference was statistically nonsignificant (40 vs. 31 %, *p* = 0.29, Fig. 6). CD68 positive histiocytes represented a minor cell component in all the cases except when siliconomas were present.

## Discussion

Aside from the morphologic features of the fibrous capsule that surrounds silicone gel breast implants, knowledge about the nature of the inflammatory response is scant. In addition to a band of dense fibrous tissue, the implant capsule includes a variable number of inflammatory cells [9]. Neutrophils are usually the first cells to arrive to the inflammatory process, digesting destroyed tissue. Monocytes, which are also identified, have phagocytic properties, becoming macrophages after engulfing any foreign substance. However, if the size of the particle to be eliminated is great, monocytes coalesce to form multinucleated giant cells. In addition to neutrophils and monocytes/macrophages, T and B lymphocytes are identified early. B cells are involved in the humoral response by producing circulating antibodies when they differentiate to plasma cells. T cells are involved in the cellular immunity. Nevertheless, the relationship between B and T lymphocytes, as well as the different proportions of T cell subtypes, has been infrequently reported in capsular implants.

Most of previously published studies, in which an immunophenotype for inflammatory cells in the capsular implants or fluid taken from the space between the implants and the surrounding fibrous capsule were determined, showed a predominance of T cells over B cells [2, 10]. Our findings are in agreement with those previous reports since the majority of inflammatory cells present in the implant capsule were T cells with a similar proportion for helper and cytotoxic subtypes. Thus, our findings support the hypothesis that silicone may induce a strong local T cell immune response. Even though the number of smooth-surfaced implants in our study was limited, they showed a lower tendency to elicit an inflammatory response. This finding would have to be confirmed by future studies. On the other hand, a variety of immunologic diseases (e.g., rheumatic diseases, including chronic rheumatoid arthritis, systemic lupus erythematosus, and Sjogren's syndrome, and the recently identified ALK-negative anaplastic large cell lymphoma) has been reported in association with breast implants [11, 12]. Of particular interest is the latter due to its clinical implications.

Primary lymphoma of the breast usually accounts for less than 1 % of all non-Hodgkin's lymphomas, and between 0.4 and 1 % of malignant breast neoplasms [13]. Epidemiologic studies assessing the risk of non-Hodgkin's lymphoma in women with breast implants found no association between breast implants and an increased risk of non-Hodgkin's lymphoma [14, 15]. However, in 1997, Keech and Crech described a case of anaplastic large cell lymphoma negative for anaplastic lymphoma kinase-1 (ALCL-ALK1 negative) associated with a breast implant. Since this first report, 35 cases of ALCL in women with breast implants have been documented worldwide, turning it in a rare but emerging entity [16].

Similar to other lymphomas reported in association with breast implants, ALCL shows, with rare exceptions, a T cell phenotype. In the present study, we documented a predominance of T cells when the inflammatory cells were present in the breast implant capsule. This predominance lead us to believe that there might exist conditions related to either the capsular component of implants or to the content of implants that could facilitate the proliferation of T cells over B cells. On the other hand, reported ALCL cases related to implants, for which a reference to the type of implants exists, have always been of textured surface. It is worth noting that even though the number of smooth surface implants in our study was limited, a T cell predominance tendency, as those seen in textured-type implants, was not observed. This fact backs up the hypothesis about smooth surfaced implants being less selective for T cells. Although the relationship between implants and these diseases is still unclear, a broader study is needed to confirm the present findings and determine its relationship with these diseases.

In summary, capsular contracture was associated with implant profile (low vs. high) but no with implant type (smooth vs. textured) or location (subglandular vs. submuscular). We also found evidence of inflammation in 92.5 % of all implant capsules, with a statistically significant higher proportion in textured breast implants. T cells predominated over B cells. Textured implants elicited a more marked response to T cells than smooth implants, with a similar proportion of helper and cytotoxic T cells. Textured implants showed statistically significant higher percentages of CD3 positive cells than smooth implants. Percentages of CD20 positive cells were similar in textured and smooth implants. These results suggest that the silicone present in breast implants might induce a strong local T cell immune response. Our findings could shed some light to understand the association of silicone breast implants and some cases of anaplastic large cell lymphomas.
